# SARS‐CoV‐2 and guttate psoriasis: A case report and review of literature

**DOI:** 10.1002/ccr3.4568

**Published:** 2021-07-16

**Authors:** Elrazi Ali, Abdelaziz Mohamed, Joud Abuodeh, Mhd Kutaiba Albuni, Najlaa Al‐Mannai, Sarah Salameh, Mahir Petkar, Elmukhtar Habas

**Affiliations:** ^1^ Internal Medicine Department Hamad Medical Corporation Doha Qatar; ^2^ College of Medicine Qatar University Doha Qatar; ^3^ Infectious Disease Department Hamad Medical Corporation Doha Qatar; ^4^ Histopathology Department Hamad Medical Corporation Doha Qatar

**Keywords:** COVID‐19, guttate psoriasis, psoriasis, SARS‐CoV‐2

## Abstract

Guttate psoriasis is a rare dermatological presentation of SARS‐CoV‐2 infection and is seen mainly in patients with an underlying disease psoriasis.

## Graphical abstract

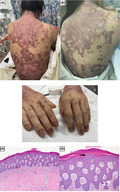

## 1 BACKGROUND

Severe acute respiratory syndrome coronavirus 2 (SARS‐CoV‐2) infection is an airborne disease with various degrees of severity ranging from asymptomatic to multiorgan failure. It often presents with non‐specific symptoms such as fever, headache, and fatigue, accompanied by respiratory symptoms (e.g. cough and dyspnea) and other systemic involvement, including the skin. Several dermatological manifestations were reported to occur with SARS‐CoV‐2. They include various types of skin rash and worsening of underlying skin disease. However, only a few cases of SARS‐CoV‐2 infection have been linked to precipitate guttate psoriasis. We report an unusual case of SARS‐CoV‐2 infection in a 30‐year‐old lady with no previous medical illness who developed severe psoriasis post‐SARS‐CoV‐2 infection. She responded well to cyclosporine. Psoriasis can be triggered by various infections, including SARS‐CoV‐2 infection. We are reporting an unusual case of the post‐SARS‐CoV‐2 infection triggering severe psoriasis.

Severe acute respiratory syndrome coronavirus 2 (SARS‐CoV‐2) infection as a novel coronavirus causes a cluster of pneumonia cases in Wuhan city in China at the end of 2019. The virus causes coronavirus disease 2019 (COVID‐19), ranging in severity from mild asymptomatic to fatal disease due to severe acute respiratory distress syndrome and respiratory failure.^1^ Hospital admission is more common in the elderly population, and mortality is high among older patients and patients with comorbidities.^2^, ^3^ It typically presents with symptoms of fever and upper respiratory symptoms. Non‐respiratory presentation of SARS‐CoV‐2 infection includes gastrointestinal symptoms such as diarrhea, vomiting, and diabetic ketoacidosis.^4^, ^5^ Moreover, SARS‐CoV‐2 was reported to present as and precipitate heart failure and even fulminant liver failure.^6^, ^7^ Among the various presentation of SARS‐CoV‐2 infection, the cutaneous manifestations were not uncommon. Psoriasis is an inflammatory skin condition characterized by well‐demarcated erythematous plaques with an overlying scale. Commonly, it is a chronic condition, but it may present as an acute eruption in patients with no previous disease or worsening of the chronic rash called guttate psoriasis.

We present a young lady with no significant past medical history presented with severe guttate psoriasis after SARS‐CoV‐2 infection. She developed guttate psoriasis and responded well to treatment.

## 2 CASE PRESENTATION

Thirty years female from Nepal with no past medical history presented on March 2021 with a low‐grade fever and mild cough. She was diagnosed with SARS‐CoV‐2 infection using fully automated reverse‐transcription polymerase chain reaction (RT‐PCR) Cobas^®^ 6800 (Roche) from nasopharyngeal and throat swabs with CT 15.45. Chest X‐ray was unremarkable apart from increased bronchovascular marking, and she was classified as mild SARS‐CoV‐2 disease according to the WHO case definition.^8^ She only received vitamin C tablets along with paracetamol during her SARS‐CoV‐2 infection. She recovered from fever and cough and tested negative 20 days later. Blood investigation is shown in Table 1.

**TABLE 1:** The table shows the blood work done, including renal function and inflammatory markers

Detail	Value w/Units	Normal range
WBC	7.3 × 10^3^/ul	4.0–10.0
Hgb	9.2 gm/dl	12.0–15.0
MCV	83.1 fl	83.0–101.0
Platelet	523 × 10^3^/ul	150–400
Lymphocyte	1.2 1.2 × 10^3^/ul	1–3
Eosinophil	0.2	0–0.5
Urea	1.7 mmol/L	2.5–7.8
Creatinine	35 umol/L	44–80
ALT	12 U/L	0–33
Vit D	14 ng/ml	More than 20
CRP	60.8 mg/L	0.0–5.0
Procalcitonin	0.03 ng/ml	<0.5 low risk of sepsis
ANA CTD Int	Negative	
Bhcg	<1 mIU/ml	0–5
Hepatitis B surface antigen	Nonreactive	
Hepatitis C Ab	Nonreactive	
HIV Ag/Ab combo	Nonreactive	

**FIGURE 1:**
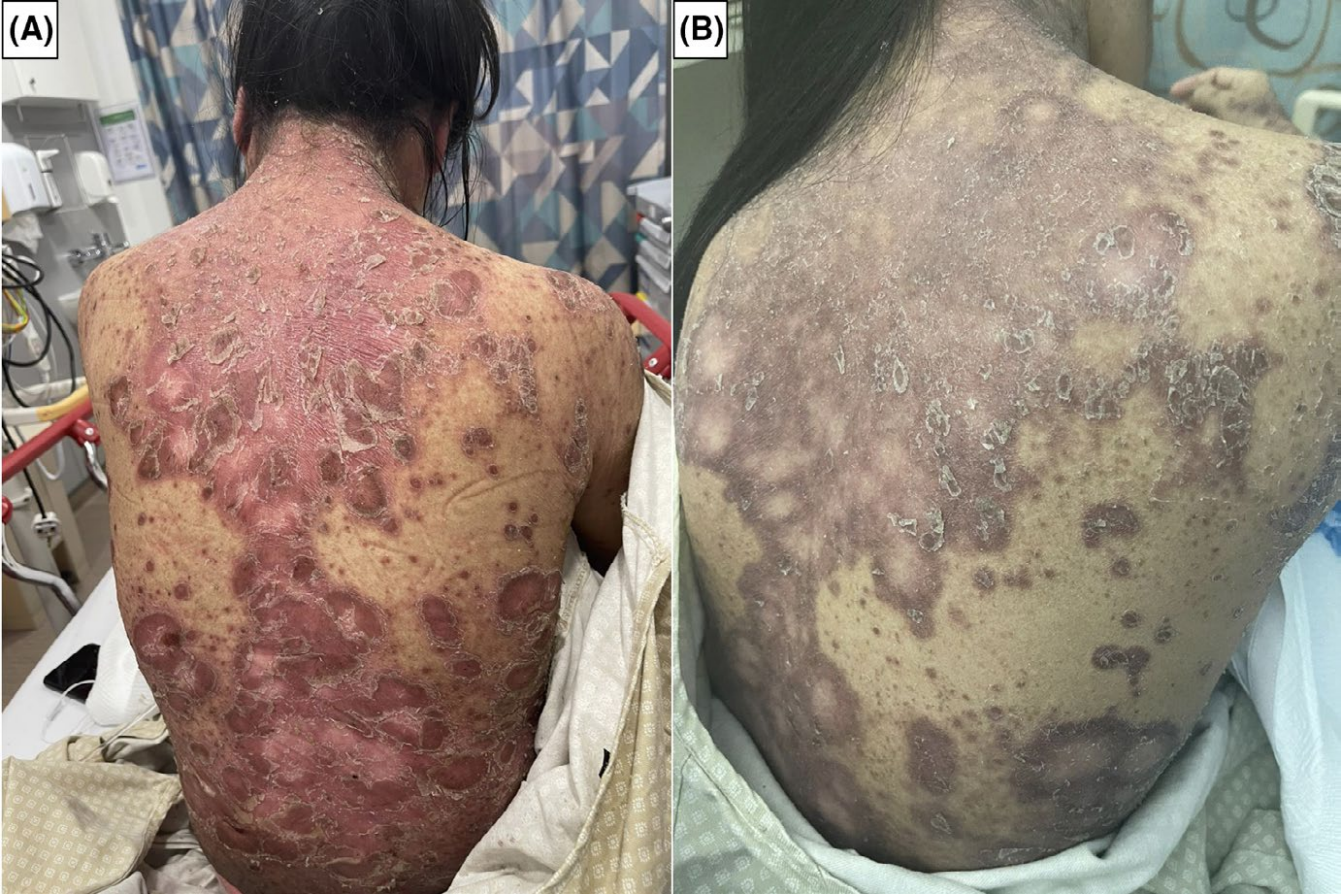
Shows the skin eruption. (A) before and (B) after treatment after cyclosporine

**FIGURE 2:**
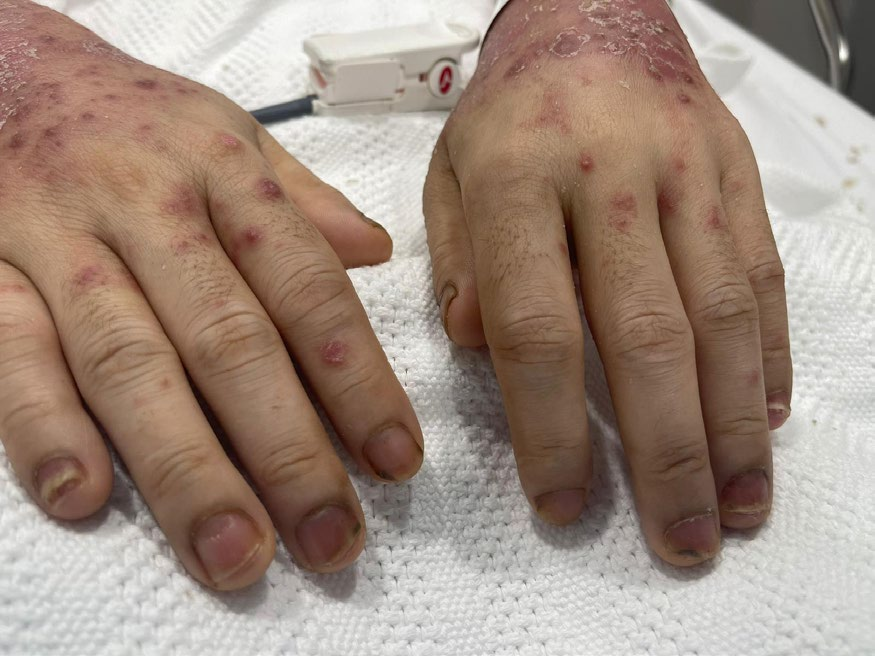
Showed nail pitting

**FIGURE 3:**
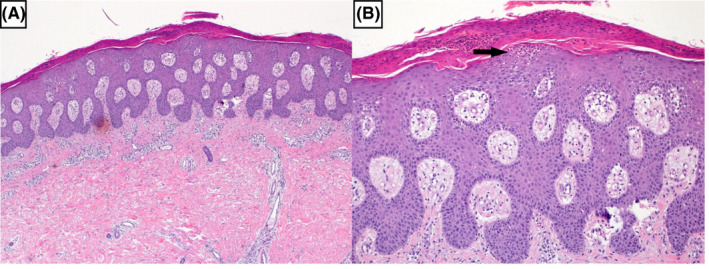
A, Psoriasiform hyperplasia with surface hyperkeratosis and parakeratosis (H and E ×20). B, Higher power view revealing Munro microabscess (black arrow) (H and E ×40)

Two days after testing positive for SARS‐CoV‐2, she noticed a painful scaly rash that progressed to involve most of her skin (Figure 1). The rash was a widespread painful, itchy erythematous skin rash with significant scaling that mainly affects the trunk, the back, upper limbs, and scalp. The rash spares her face, palms, and soles. She first noticed a painless scattered slightly scaly rash with nail changes 6 months ago( Figure 2), which she did not seek medical attention. The lesions were stable until the presentation when she noticed a rapid progression of the rash. There were no associated joint pain or swelling; she has no family history of similar conditions, and she did not use any topical creams other than Vaseline. Her vitals showed one episode of Fever spiking to 39 degrees, and blood pressure was on the lower borderline of 100–90 SBP with tachycardia in the range of 90–110 beats per minute. Bodyweight was 45 kg. On examining the patient, scaly, well‐demarcated erythematous plaques were noticed over the trunk, limbs, scalp, and ears covering approximately 60% of body surface area. Mouth examination was unremarkable no erythema or congestion. Skin punch biopsy from the abdomen revealed prominent regular acanthosis with elongated rete (psoriasiform hyperplasia), as well as surface hyperkeratosis and parakeratosis. The granular layer was lost. Foci of neutrophilic microabscesses were noted within the stratum corneum (Munro microabscesses). The superficial dermis displayed perivascular predominantly lymphocytic inflammatory infiltrate with scattered neutrophils. Deep dermis was unremarkable. Fungal stain was negative. Histologically, the appearances were in keeping with psoriasis (Figure 3 ).

She was admitted for 5 days during she received generous IV hydration and started on oral Cyclosporine 100 mg twice daily therapy (after SARS‐CoV‐2 was negative), along with oral minocycline, topical mometasone 1% cream with emollients. The renal function was stable. Her skin rash improved significantly, and she was discharged on cyclosporine and mometasone cream with dermatology follow‐up.

## 3 DISCUSSION AND CONCLUSION

SARS‐CoV‐2 is a common infection during the last 18 months. Various presentations are noticed; among these different presentations is the cutaneous manifestation. Dermatological manifestation of SARS‐CoV‐2 was reported in the case series. Skin involvement in SARS‐CoV‐2 infection is not uncommon: Approximately 0.2% to 20.4% of SARS‐CoV‐2 infected patients had skin lesions.^9^ The timing of cutaneous manifestations of SARS‐CoV‐2 is difficult to predict; it was reported at the onset of the illness and event during recovery.^9^ There is no clear link between the severity of SARS‐CoV‐2 and the presence of cutaneous manifestation.^10^ Various types of skin rash were reported; the most common was exanthematous (morbilliform) rash. The morbilliform rash predominantly involves the trunk and more frequently reported after hospital discharge or recovery.^9^ Other types of skin rash associated with SARS‐CoV‐2 infection include Pernio (chilblain)‐like acral lesions, vesicular (varicella‐like) eruptions, urticaria, livedo reticularis‐like, and fixed livedo racemosa/retiform purpura. Interestingly, retiform purpura lesions seem to be associated with severe SARS‐CoV‐2.^11^

Psoriasis is not an uncommon skin disease; it has a relatively high prevalence in the general population, estimated to be around 0.6% to 4.8%.^12^ The most common form of psoriasis is chronic plaque psoriasis. Guttate psoriasis is a form of psoriasis that presents acutely and is commonly seen in children and young adults under 30, but all age groups can be affected.^13^ Typically, guttate psoriasis occurs following acute infections, most commonly streptococcal infection seen in more than 50% of patients.^14^ The exact pathophysiology of guttate psoriasis is not well understood. The strong link to streptococcal infection proposed that the underlying mechanism is related to cross‐reactivity between streptococcal M‐proteins or molecular mimicry.^15^ Additional mechanisms include streptococcal super‐antigens acting to augment the expression of the skin‐homing cutaneous lymphocyte antigen (CLA) on effector T cells, contributing to the migration of T cells into the skin. Addition theory is an innate immune‐mediated inflammatory response that occurs as a consequence of the binding of peptidoglycan or other streptococcal antigens to cell receptors.

SARS‐CoV‐2 infection was reported to trigger attacks of psoriasis. However, SARS‐CoV‐2 infection was rarely reported to trigger guttate psoriasis. A review of the literature showed only one case report who developed guttate psoriasis following SARS‐CoV‐2.^16^ The reported patient^16^ had a history of chronic plaque psoriasis, which was not active, and developed the skin rash on day 6 after the fever's onset. The skin lesions were not severe, and the patient was treated with betamethasone 0.025% cream and did not require immunosuppressive medications. Our patient has mild skin changes suggestive of psoriasis, and it was so mild that she did not seek medical evaluation for it. Additionally, there was nail pitting (Figure 2 ) which indicates that the patient had mild chronic plaque psoriasis that was unnoticed and SARS‐CoV‐2 lead to trigger the flare as guttate psoriasis. The skin rash flared two days after the onset of SARS‐CoV‐2 and continued to worsen during the recovery from SARS‐CoV‐2. The respiratory symptoms were mild, the chest X‐ray was unremarkable, and she did not develop any thrombotic complications.^17^ Despite that, she had severe skin eruption supporting the reported fact that skin rash with SARS‐CoV‐2 has no relation to the severity of the respiratory symptoms. The SARS‐CoV‐2 virus may cause the eruption of guttate psoriasis by a similar mechanism to streptococcal infection by molecular mimicry, or the cytokine excess might cause it during the acute infection.^15^ Similar findings were observed with other viral infections like^18^ SARS‐CoV‐2 virus is not commonly causing guttate psoriasis compared to other cutaneous manifestations, the reason is mostly related to the genetic predisposition as with psoriasis is known to be higher in people with specific HLA type like HLA‐Cw*0602.^19^ Furthermore, no cases of guttate psoriasis were reported to occur in patients without pre‐existing psoriasis, indicating that the main factor is the genetic predisposition. The patient received cyclosporine with significant improvement in few days (Figure 1); during that time, she was tested negative on RT‐PCR. The difficulty in starting treatment in patients with SARS‐CoV‐2 on immunosuppressive medication is the increased risk of infection and particularly with cyclosporine, which is associated with increased BP, which worsens the outcome from SARS‐CoV.^20^

In conclusion, SARS‐CoV‐2 can have various skin manifestations: Of these manifestations, guttate psoriasis is a rare finding. It is seen in patients with the chronic form of psoriasis not reported in patients without previous skin disease. Our patient responded well to cyclosporine therapy, but caution should be taken if the patient is still SARS‐CoV‐2 positive.

## 4 ETHICS

The case report was approved by the Medical Research Center with MRC‐04–21–358.

## 5 CONSENT

Written informed consent was obtained from the patient for publication of this case report.

## CONFLICT OF INTEREST

All authors have no conflict of interest.

## AUTHOR CONTRIBUTION

Elrazi Ali, Abdelaziz Mohamed, Joud Abuodeh, Mohamed Albuni, Najlaa Al‐Mannai, Sarah Salameh, Mahir Petkar, and Elmukhtar Habas were involved in writing and editing the final approval of the manuscript.

## Data Availability

Data are available on reasonable request.

## References

[1] Wang D, Hu B, Hu C, et al. Clinical characteristics of 138 hospitalized patients with 2019 novel coronavirus‐infected pneumonia in Wuhan, China. JAMA. 2020;323(11):1061‐1069. 32031570 10.1001/jama.2020.1585 PMC7042881

[2] Iqbal F, Soliman A, De Sanctis V, et al. Prevalence, clinical manifestations, and biochemical data of hypertensive versus normotensive symptomatic patients with COVID‐19: a comparative study. Acta Bio Medica: Atenei Parmensis. 2020;91(4). 10.23750/abm.v91i4.10540 PMC7927505 33525211

[3] Soliman A, Nair AP, Al Masalamani MS, et al. Prevalence, clinical manifestations, and biochemical data of type 2 diabetes mellitus versus nondiabetic symptomatic patients with COVID‐19: A comparative study. Acta Bio Medica: Atenei Parmensis. 2020;91(3):e2020010. 10.23750/abm.v91i3.10214 PMC7716959 32921708

[4] Ali E, Badawi M, Ahmed A, Abdelmahmuod E, Ibrahim W. Severe SARS‐CoV‐2 infection presenting with acute kidney injury and diabetic ketoacidosis complicated by pancreatitis in a 53‐year man with hypertension. Clin Case Rep. 2021;9(3):1202‐1206. 33768811 10.1002/ccr3.3731 PMC7981639

[5] Ata F, Almasri H, Sajid J, Yousaf Z. COVID‐19 presenting with diarrhoea and hyponatraemia. BMJ Case Rep. 2020;13(6):e235456. 10.1136/bcr-2020-235456 PMC10577739 32513768

[6] Ata F, Montoro‐Lopez MN, Awouda S, Elsukkar AM, Badr AM, Patel AA. COVID‐19 and heart failure: the big challenge. Heart Views. 2020;21(3):187. 33688410 10.4103/HEARTVIEWS.HEARTVIEWS_122_20 PMC7898990

[7] Ali E, Ziglam H, Kohla S, Ahmed M, Yassin M. A case of fulminant liver failure in a 24‐year‐old man with coinfection with hepatitis B virus and SARS‐CoV‐2. Am J Case Rep. 2020;21:e925932‐e925941. 33046686 10.12659/AJCR.925932 PMC7568525

[8] Clinical management of COVID‐19. https://www.who.int/publications/i/item/clinical‐management‐of‐covid‐19

[9] Recalcati S. Cutaneous manifestations in COVID‐19: a first perspective. J Eur Acad Dermatol Venereol. 2020;34(5):e212‐e213. 32215952 10.1111/jdv.16387

[10] Suchonwanit P, Leerunyakul K, Kositkuljorn C. Cutaneous manifestations in COVID‐19: lessons learned from current evidence. J Am Acad Dermatol. 2020;83(1):e57‐60. 32339706 10.1016/j.jaad.2020.04.094 PMC7194618

[11] Freeman EE, McMahon DE, Lipoff JB, et al. The spectrum of COVID‐19–associated dermatologic manifestations: an international registry of 716 patients from 31 countries. J Am Acad Dermatol. 2020;83(4):1118‐1129. 32622888 10.1016/j.jaad.2020.06.1016 PMC7331510

[12] Naldi L. Epidemiology of psoriasis. Curr Drug Targets Inflam Allergy. 2004;3(2):121‐128. 10.2174/1568010043343958 15180464

[13] Prinz JC. Psoriasis vulgaris–a sterile antibacterial skin reaction mediated by cross‐reactive T cells? An immunological view of the pathophysiology of psoriasis. Clin Exp Dermatol. 2001;26(4):326‐332. 11422184 10.1046/j.1365-2230.2001.00831.x

[14] Ko H‐C, Jwa S‐W, Song M, Kim M‐B, Kwon K‐S. Clinical course of guttate psoriasis: long‐term follow‐up study. J Dermatol. 2010;37(10):894‐899. 20860740 10.1111/j.1346-8138.2010.00871.x

[15] Valdimarsson H, Thorleifsdottir RH, Sigurdardottir SL, Gudjonsson JE, Johnston A. Psoriasis–as an autoimmune disease caused by molecular mimicry. Trends Immunol. 2009;30(10):494‐501. 19781993 10.1016/j.it.2009.07.008

[16] Gananandan K, Sacks B, Ewing I. Guttate psoriasis secondary to COVID‐19. BMJ Case Rep. 2020;13(8):e237367. 10.1136/bcr-2020-237367 PMC7418771 32784237

[17] Mohamed MF, Al‐Shokri SD, Shunnar KM, et al. Prevalence of venous thromboembolism in critically Ill COVID‐19 patients: systematic review and meta‐analysis. Front Cardiovasc Med. 2020;7. 10.3389/fcvm.2020.598846 PMC7874113 33585578

[18] Sbidian E, Madrange M, Viguier M, et al. Respiratory virus infection triggers acute psoriasis flares across different clinical subtypes and genetic backgrounds. Br J Dermatol. 2019;181(6):1304‐1306. 31150103 10.1111/bjd.18203 PMC7161746

[19] Fan X, Yang S, Sun LD, et al. Comparison of clinical features of HLA‐Cw* 0602‐positive and‐negative psoriasis patients in a Han Chinese population. Acta Dermato‐Venereol. 2007;87(4):47‐52. 10.2340/00015555-0253 17598037

[20] Elmas ÖF, Demirbaş A, Kutlu Ö, et al. Psoriasis and COVID‐19: a narrative review with treatment considerations. Dermatol Ther. 2020;33(6):e13858. 32686245 10.1111/dth.13858 PMC7323009

